# Deep Neck Space Infections: A Retrospective Study of 183 Cases at a Tertiary Hospital

**DOI:** 10.7759/cureus.6841

**Published:** 2020-02-01

**Authors:** Dakheelallah Almutairi, Raneem Alqahtani, Noorah Alshareef, Yousef S Alghamdi, Hadi Afandi Al-Hakami, Mohammed Algarni

**Affiliations:** 1 Otolaryngology - Head and Neck Surgery, King Saud Bin Abdulaziz University for Health Sciences, King Abdullah International Medical Research Center, King Abdulaziz Medical City, Ministry of National Guard Health Affairs, Jeddah, SAU; 2 Medicine, College of Medicine, King Saud Bin Abdulaziz University for Health Sciences, Jeddah, SAU; 3 Otolaryngology, King Saud Bin Abdulaziz University for Health Sciences, King Abdulaziz Medical City, King Abdullah International Medical Research Center, Jeddah, SAU; 4 Otolaryngology - Head and Neck Surgery, King Saud bin Abdulaziz University for Health Sciences, King Abdullah International Medical Research Center, Ministry of National Guard Health Affairs, Jeddah, SAU; 5 Otolaryngology - Head and Neck Surgery, King Saud Bin Abdulaziz University for Health Sciences, King Abdulaziz Medical City, Jeddah, SAU

**Keywords:** deep neck space abscess, infections, neck, dental infection, dnsi, ludwig’s angina

## Abstract

Objective

Our study was performed to identify the clinical findings, risk factors, and complications of deep neck space infections (DNSI) at our center and compare our experience with the experiences of others.

Methods

Retrospectively, 183 cases of DNSI met our inclusion criteria from 2000 to 2018 at King Abdulaziz Medical City (KAMC) in Jeddah, Western Region, Saudi Arabia.

Results

In our study, analysis showed that males are more likely to have DNSI (88.7%). The most common site of infection is the peritonsillar abscess (30.6%). Dental infections were found to be the most common etiological factor for DNSI (42.6%). Streptococcus pyogenes was found to be the most common microorganism (39.3%) followed by Staphylococcus aureus (21.3%). Diabetes and hypertension (45.2% and 23.7%, respectively) are the most commonly associated disorders in patients with DNSI. Extension to another space was the most common complication of DNSI.

Conclusion

Despite the wide usage of antibiotics, DNSI still occur and are life-threatening conditions that need urgent management to avoid unpleasant complications.

## Introduction

Deep neck space infections (DNSI) are infections in the facial planes and spaces of the neck. They project a severe clinical challenge and they still are relevant health issues although antibiotics have reduced the likelihood of becoming so prevalent. The complicated anatomic framework of the neck makes the process of diagnosis difficult. Hence, clinical suspicion is essential during the diagnosis since several deep neck infections are not evident on palpation [1]. Delays in countering the infection may result in riskier issues, such as pneumonia, arterial erosion, and mediastinal involvement, even with the incorporation of modern antibiotics. Initially, 70% of the infections developed from tonsillitis before the introduction of antibiotics. Though tonsillitis remains to be the most significant cause of the disease among children, dental infections are the most prevalent causes of deep neck infections among adults [2]. To counter the infection, otorhinolaryngologists should have appropriate knowledge of presentation, etiology, investigation, and effective medical and surgical interventions. Up until current knowledge, there is limited published data about DNSI in Saudi and the primary objective of this research is to project experience on the presentation, complications, results, and clinical trends of DNSI at our center and compare it with other literature.

## Materials and methods

This is a descriptive retrospective study design conducted at King Abdulaziz Medical City (KAMC) in Jeddah, Western Region, Saudi Arabia. The study populations are all pediatric and adult patients who were clinically or radiologically confirmed cases of deep neck space infections (DNSI) from 2000 to 2018. Patients with a superficial skin abscess or a limited-space intraoral abscess were excluded from the study. A total of 183 cases of DNSI met our inclusion criteria. A datasheet that has the parameters - age, gender, co-morbidities, symptoms, site involved, bacteriology, culture growth, type of intervention required, complications, and outcome - was used. All patients received empirical treatment, alone or in combination, of ceftriaxone, clindamycin, and metronidazole; later on, the treatment regimen was changed based on the culture and sensitivity report. Initial hematological and laboratory investigations were done for all patients, including complete blood count (CBC), erythrocyte sedimentation rate (ESR) and serum creatinine. In some cases where the suspicion of tuberculous lymphadenopathy was high, Mendel-Mantoux was performed. Other specific investigations, such as fine-needle aspiration cytology, ultrasonography and X-rays, computed tomography (CT), and magnetic resonance imaging (MRI), were done wherever needed. Approval from the Regional Research and Ethics Committee at King Abdullah International Medical Research Center Western Region was obtained, and ethical considerations were taken through all research steps. Data were entered into the workplace computer by the researchers and were analyzed using the Statistical Package of the Social Sciences (SPSS) version 25 (IBM Corp, Armonk, NY).

## Results

The study group of patients with DNSI comprised 183 patients: 162 males (88.5%) and 21 females (11.5%). Patients ranging in age from birth to 70 years and above were included (median 47). The majority of the patients were seen in the fifth decade of life (24%), which is followed by the sixth decade (20.7%) as shown in Table 1.

**Table 1:** Distribution of gender and age in patients with DNSI (n=183) DNSI: deep neck space infection

Distribution of gender and age in patients with DNSI (n=183)		
Gender	Number of patients	Percentages
Male	162	88.5%
Female	21	11.5%
Age (years)	Number of patients	Percentages
1-10	16	8.7%
11-20	11	6%
21-30	14	7.7%
31-40	20	11%
41-50	44	24%
51-60	38	20.7%
61-70	30	16.4%
>70	10	5.5%
Total	183	100%

Table 2 shows common symptoms in patients with DNSI. The most common symptom was pain, noted in 109 patients (59.6%), dysphagia was observed in 80 patients (43.7%), toothache was prevalent in 78 patients(42.6%), odynophagia was present in 56 patients (30.6%), sore throat in 43 patients (23.4%), 35 patients (19.3%) had symptoms of otalgia, airway obstruction was prevalent in 25 patients (13.7%), hoarseness was noted in 18 patients (9.8%), 16 patients (8.7%) complained of headache, 14 patients (7.6%) had a cough, and six patients (3.3%) had back pains.

**Table 2:** Symptoms and signs in patients with DNSI (n=183) DNSI: deep neck space infection

Symptoms and signs in patients with DNSI (n=183)		
Symptoms	Number of patients	Percentages
Pain	109	59.6%
Dysphagia	80	43.7%
Toothache	78	42.6%
Odynophagia	56	30.6%
Sore throat	43	23.4%
Otalgia	35	19.3%
Airway obstruction	25	13.7%
Hoarseness	18	9.8%
Headache	16	8.7%
Cough	14	7.6%
Back pain	6	3.3%

As seen in Table 3, patients with DNSI had the following presenting signs: neck swelling was observed in 140 patients (76.5%), 102 patients (59.6%) had fever, trismus was prevalent in 34 patients (18.6%), 32 patients (17.5%) had rancid breath, sialorrhea was noted in 31 patients (17%), 16 patients (8.7%) experienced gingival swelling, and 11 patients (6%) had a muffled voice.

**Table 3:** Presenting signs in patients with DNSI (n=183) DNSI: deep neck space infection

Presenting signs in patients with DNSI (n=183)		
Signs	Number of patients	Percentages
Neck swelling	140	76.5%
Fever	102	55.7%
Trismus	34	18.6%
Rancid breath	32	17.5%
Sialorrhea	31	17%
Gingival swelling	16	8.7%
Muffled voice	11	6%

Etiological factors present in patients with DNSI can be seen in Table 4. Odontogenic was the most common, as it was noted in 78 patients (42.6%), tonsillopharyngitis was observed in 49 patients (26.8%), 26 patients (14.2%) had infected cysts, and 13 patients (7.1%) had an unknown etiology. Salivary gland infections were complained by eight patients (4.4%), seven patients (3.9%) had tuberculous lymphadenopathy, and the mandibular fracture was noted in two patients (1%).

**Table 4:** Etiological factors in patients with DNSI (n=183) DNSI: deep neck space infection

Etiological factors in patients with DNSI (n=183)		
Etiological factor	Number of patients	Percentages
Odontogenic	78	42.6%
Tonsillopharyngitis	49	26.8%
Infected cysts	26	14.2%
Unknown	13	7.1%
Salivary gland infections	8	4.4%
Tuberculous lymphadenopathy	7	3.9%
Mandibular fracture	2	1%

The location of abscesses in patients with DNSI was experienced in different parts as seen in Table 5. Fifty-six patients (30.6%) had peritonsillar abscess, 34 patients (18.6%) had submandibular abscess, 32 patients (17.5%) experienced parapharyngeal abscess, Ludwig's angina was noted in 14 patients (7.7%), 14 patients (7.7%) had an anterior triangle neck abscess while 10 patients (5.2%) had retropharyngeal abscess, parotid abscess was seen in 10 patients (5.2%), prevertebral abscess was prevalent in eight patients (4.3%), and five patients (2.6%) had a posterior triangle neck abscess.

**Table 5:** Locations of abscesses in patients with DNSI (n=183) DNSI: deep neck space infection

Locations of abscesses in patients with DNSI (n=183)		
Location	Number of patients	Percentages
Peritonsillar abscess	56	30.6%
Submandibular abscess	34	18.6%
Parapharyngeal abscess	32	17.5%
Ludwig’s angina	14	7.7%
Anterior triangle neck abscess	14	7.7%
Retropharyngeal abscess	10	5.5%
Parotid abscess	10	5.5%
Prevertebral abscess	8	4.3%
Posterior triangle neck abscess	5	2.6%

Table 6 shows bacteriology in DNSI patients. Streptococcus pyogenes was most common, as it was found in 72 patients (39.3%), Staphylococcus aureus was discovered in 39 patients (21.3%), Streptococcus variance was present in 17 patients (9.3%), Peptostreptococcus was present in 15 patients, 11 patients (5.7%) had Bacteroides, Mycobacterium tuberculosis was found in seven patients (3.6%), six patients (3.1%) had negative cultures, Pseudomonas aeruginosa was identified in four patients (2.1%), and Escherichia coli was observed in four patients (2.1%), and, lastly, Streptococcus pneumoniae was found in three patients (1.7%).

**Table 6:** Bacteriology in patients with DNSI (n=183) DNSI: deep neck space infection

Bacteriology in patients with DNSI (n=183)		
Bacteriology	Number of patients	Percentages
Streptococcus pyogenes	72	39.3%
Staphylococcus aureus	39	21.3%
Streptococcus viridans	17	9.3%
Peptostreptococcus	15	8.2%
Bacteroides	11	6%
Mycobacterium tuberculosis	7	3.9%
Negative culture	6	3.3%
Klebsiella pneumonia	5	2.6%
Pseudomonas aeruginosa	4	2.2%
Escherichia coli	4	2.2%
Streptococcus pneumoniae	3	1.7%

Various methods were used to manage the different cases of DNSI patients. These can be seen in Table 7. Incision and drainage were the most preferred, as 87 patients (47.5%) had their case managed by this method. Incision, drainage, and teeth extraction was the method used to handle 78 patients (42.7%). Six patients (3.3%) underwent incision, drainage, and debridement, five patients (2.6%) underwent incision, drainage, and tracheostomy, four patients (2.2%) had an incision, drainage, and antitubercular therapy performed on them, and antitubercular therapy was used to manage three patients (1.7%). Antitubercular therapy included a multidrug regimen of first-line drugs: isoniazid (INH), rifampin (RIF), pyrazinamide (PZA), ethambutol (EMB), and streptomycin (SM) for six months.

**Table 7:** Management in patients with DNSI (n=183) DNSI: deep neck space infection

Management in patients with DNSI (n=183)		
Management	Number of patients	Percentages
Incision and drainage	87	47.5%
Incision, drainage, and teeth extraction	78	42.7%
Incision, drainage, and debridement	6	3.3%
Incision, drainage, and tracheostomy	5	2.6%
Incision, drainage, and antitubercular treatment	4	2.2%
Antitubercular treatment	3	1.7%

Other disorders were noted in patients with DNSI, as summarized in Table 8. Ninety-three out of the 183 patients had diseases, 42 patients (45.2%) suffered from diabetes, 22 patients had hypertension (23.7%), heart disease was a disorder common in nine patients (9.7%) eight patients had pneumopathy (8.5%), rheumatic disease was prevalent in seven patients, and thyroid disorder was noted in five patients (7.5%).

**Table 8:** Associated disorders in patients with DNSI (n=183) DNSI: deep neck space infection

Associated disorders in patients with DNSI (n=183)		
Disorders	Number of patients	Percentages
Diabetes	42	45.2%
Hypertension	22	23.7%
Heart disease	9	9.7%
Pneumopathy	8	8.5%
Rheumatic disease	7	7.5%
Thyroid disorder treatment	5	5.4%

In this research, patients with DNSI experienced some complications, as seen in Table 9. Out of the 194 patients, only 38 had these complications. Extension to another space was identified in 12 patients (31.5%), septicemia was found in 15 patients (39.5%), six patients (15.8%) were diagnosed with pneumonia, and airway obstruction was noted in five patients (13.2%).

**Table 9:** Complications in patients with DNSI (n=183) DNSI: deep neck space infection

Complications in patients with DNSI (n=183)		
Complication	Number of patients	Percentages
Septicemia	15	39.5%
Extension to another space	12	31.5%
Pneumonia	6	15.8%
Airway obstruction	5	13.2%

## Discussion

Although DNSI can affect people of all ages, in our research, people aged 41-60 years were the most affected while persons above 70 years were the least affected. A study by Gujrathi et al. found that the majority of their patients were in the third decade (21.85%) followed by the fourth decade of life (18.15%) [3]. Results by Har-El et al. also showed that almost 50% of their patients were in their thirties or forties [4]. Al-Noury et al.'s observations seemed to correspond with ours whereby, in their study, the mean age for infections was 43.3 years [5].

The most common symptom in our patients was pain, complained of by 59.6% of our patients, followed by dysphagia and toothache at 43.7% and 42.6%, respectively. Kamath et al. recorded dysphagia as the most complained-about symptom, accounting for 66% of patients, followed by neck pain and neck swelling at 59% each [6]. A study by Bottin et al. showed similar results to ours where the majority of their patients complained of neck pain (71.1%), followed by odynophagia (54.2%) and dysphagia (51.8%) [7]. Pain was also the most commonly reported symptom in Gujrathi et al, accounting for 81.48%, followed by swelling 77.78% [3].

Kamath et al. found most of the patients (79%) in their study had neck swellings, with oropharyngeal abnormalities being present in 62% of the patients [6]. Neck swelling was also a major sign present in 80.7% of the patients in a study by Bottin et al., followed by fever 48.2% and trismus 24.1% [7]. our studies were consistent with those of Kamath et al. and Bottin et al., with neck swelling being present in the majority of the patients (76.5%). Our study resembled Bottin’s more, with fever being the second-most common sign (55.7%) followed by trismus (18.6%).

Our study showed that the most common etiological factor was odontogenic (42.6%), followed by tonsillopharyngitis (26.8%). This was similar to findings by Har-El et al., where (43%) was odontogenic, however, pharyngotonsillitis came third at 6.7%, after drug abuse [4]. Kamath et al. reported in their study that the sites of origin of DNSI were unknown (38%), with (28%) being odontogenic and (24%) being tonsils/pharynx infection [6]. Gujrathi et al. found that dental (42.2%) was the major site of origin, followed by pharynx (14.5%) and salivary glands (13.2%) [3]. Seventy percent of deep neck infections in the pre-antibiotic era was as a result of spread from pharyngeal and tonsillar infections while dental infections were the cause in only 20% of DNSI patients. In the post-antibiotic era, the number of infections was increasingly odontogenic and usually involved the submandibular and parapharyngeal spaces [8].

Most patients in our study had peritonsillar abscesses (30.6%) with submandibular and parapharyngeal abscesses accounting for 18.6% and 16.5% of the cases, respectively. Our study contrasted with Har-El et al., where they found a submandibular abscess in 36% of their patients, a pharyngeal abscess in 30%, and Ludwig’s abscess in 21% [4]. Kamath et al. observed most abscesses in the parapharyngeal space (48%), followed by the submandibular space (31%) and the retropharyngeal space (24%) [6]. Historically, retropharyngeal space infections were most common in children less than 10 years old. However, an increase in the immunocompromised patient population has seen these infections become more frequent in adults, and according to Al-Noury and Lotfy, the infections are more common in males than females (2:1). Patients with retropharyngeal space infections also had fever, odynophagia, dysphagia, sore throat, pains, and stiffness [5].

In our study, the most common causative agents were found to be Streptococcus pyogenes present in 39.3% of the patients and Staphylococcus aureus (21.3%). Similar to our findings, Kamath observed Streptococcus pyogenes (11/29) as the most common bacteria, however, Klebsiella (8/29) was more common than Staphylococcus aureus (2/29) [6]. Har-El et al. found Streptococcus viridans as the most common (39%), followed by Staphylococcus epidermidis (28%) and Staphylococcus aureus (22%) [4]. Al-Noury et al. found Staphylococcus aureus (25%) as the most common organism, followed by Klebsiella pneumonia [5]. The presence of low tissue oxygen tension in the loose areolar tissue of the cervical spaces favors the synergistic growth of these bacteria.

The patients in our study responded well to incision as the preferred mode of management for DNSI. Incision and drainage was the most common method (47.5%), followed by incision, drainage, and teeth extraction (42.7%). This was similar to the study by Gujrati et al. where 54.81% underwent incision and drainage and 24.07% had an incision, drainage, and teeth extraction [3]. Using a needle to drain an abscess reduces the morbidity of open surgery by limiting surgical trauma., minimizing the risk of contaminating surrounding healthy tissue, and reducing healing time.

Our study also found that diabetes and hypertension (45.2% and 23.7%, respectively) are the most commonly associated disorders in patients with DNSI. Gujrati et al. found diabetes too as the main disorder in DNSI patients, accounting for 36.30% cases followed by hypertension (24.44%) [3]. Kauffmann et al. found cardio/pulmonary diseases in 43.0% of their patients followed by diabetes mellitus in 19.0% [1]. Diabetes has been reported by several authors as a major risk factor for infection-related mortality and morbidity [5,9-10]. Chronic smoking and drinking were the main addictions in patients with DNSI (39.63% and 24.81%) according to Gujrati et al. [3].

Most patients in our study were diagnosed with septicemia (39.5%), followed by extension to another space (31.5%), Pneumonia (15.8%), and airway obstruction (13.2%). In comparison, Boscolo-Rizzo et al. found airway obstruction in 8.5% of their patients, followed by septicemia (6.0%), and pneumonia as the fourth most diagnosed case (3.3%) after descending mediastinitis (4.4%) [11].

## Conclusions

DNSI are potentially life-threatening conditions, and they still occur despite the use of antibiotics. In our study, DNSI have shown a male preponderance and are more common in the fourth and fifth decades of life. A peritonsillar abscess is the commonest DNSI. Immunocompromised patients and those with comorbidities such as diabetes mellitus and heart diseases tend to have a severe presentation, prolonged course of management, and high rate of complications. For appropriate diagnosis and management, CT is the initial appropriate imaging modality to use in cases of DNSI, especially in cases of parapharyngeal or retropharyngeal space infection. Antibiotic coverage should include gram-positive, gram-negative, and anaerobic microorganisms. In cases where an odontogenetic source of infection is established, early removal is recommended. Incision and drainage is a successful and cost-effective way of treating DNSI. The early detection and management of DNSI can decrease undesirable consequences, especially in immunocompromised patients because they need aggressive management.
