# Correction: Deep Neck Space Infections: A Retrospective Study of 183 Cases at a Tertiary Hospital

**DOI:** 10.7759/cureus.c29

**Published:** 2020-03-30

**Authors:** Dakheelallah Almutairi, Raneem Alqahtani, Noorah Alshareef, Yousef S Alghamdi, Hadi Afandi Al-Hakami, Mohammed Algarni

**Affiliations:** 1 Otolaryngology, King Saud Bin Abdulaziz University for Health Sciences, King Abdullah International Medical Research Center, King Abdulaziz Medical City, Ministry of National Guard Health Affairs, Jeddah, SAU; 2 Medicine, College of Medicine, King Saud Bin Abdulaziz University for Health Sciences, Jeddah, SAU; 3 Otolaryngology, King Saud Bin Abdulaziz University for Health Sciences, King Abdulaziz Medical City, King Abdullah International Medical Research Center, Jeddah, SAU; 4 Otolaryngology, King Saud bin Abdulaziz University for Health Sciences, King Abdullah International Medical Research Center, Ministry of National Guard Health Affairs, Jeddah, SAU; 5 Otolaryngology, King Saud Bin Abdulaziz University for Health Sciences, King Abdulaziz Medical City, Jeddah, SAU

Two typos were found in the Results section. The original, incorrect version has been replaced with the corrected version:

**Original, incorrect:** “The majority of the patients were seen in the fourth decade of life (24%), which is followed by the fourth decade (20.7%) as shown in Table 1”

**New, correct:** “The majority of the patients were seen in the fifth decade of life (24%), which is followed by the sixth decade (20.7%) as shown in Table 1”

